# 893. Myocarditis and Pericarditis as a Complication of Mpox: An International Case Series and Literature Review

**DOI:** 10.1093/ofid/ofad500.938

**Published:** 2023-11-27

**Authors:** Sheliza Halani, Sean Cai, Juan Carlos Monge, Philippe Brouillard, Cécile Tremblay, Jose Luis Blanco, Ana Isabel Pinho, Guillermo Rodriguez-Nava, Supriya Narasimhan, Joseph Cooper, Peter Kadlecik, Darrell H S Tan

**Affiliations:** University of Toronto, Toronto, Ontario, Canada; University of Toronto, Toronto, Ontario, Canada; St. Michael’s Hospital, Toronto, Ontario, Canada; Département de Médecine, Université de Montréal and Centre Hospitalier de l′Université de Montréal (CHUM), Montreal, Quebec, Canada; Infectiologie et Immunologie, Faculté de Médecine, Université de Montréal, Montreal, Quebec, Canada; University of Barcelona, Hospital Clinic-IDIBAPS, Barcelona, Catalonia, Spain; São João University Hospital Centre, Porto, Porto, Portugal; Stanford University School of Medicine, Palo Alto, California; Santa Clara Valley Medical Center, San Jose, California; Santa Clara Valley Medical Center, San Jose, California; Mid-Atlantic Permanente Medical Group, Rockville, Maryland; St. Michael’s Hospital, Toronto, Ontario, Canada

## Abstract

**Background:**

Mpox virus was declared a public health emergency of international concern by the World Health Organization in July 2022. The full range of clinical manifestations of this emerging infectious disease continues to be elucidated.

**Methods:**

We present a case series and literature review of patients with mpox myocarditis and pericarditis, including demographics, clinical symptoms, diagnostic and management strategies, and outcomes.

**Results:**

We identified 13 patients aged 21-51 (median 32) years with polymerase chain reaction-confirmed mpox and myopericarditis (n=3), pericarditis (n=1), or myocarditis (n=9), from 6 countries on 3 continents. All but one were men. One was HIV-positive (viral load undetectable) and 4 were on HIV pre-exposure prophylaxis. None had prior cardiac disease and 3 used tobacco. Most acquired mpox via sexual contact; one heterosexual patient reported non-sexual close contact. Cutaneous/mucosal lesions occurred in 11/13 patients, and fever in 11/13. Where reported, cardiac symptom onset was 2-8 (median 5.5) days after mpox illness onset. C-reactive protein ranged from 9.3-154.5 (median 52.6) mg/L. Diagnosis of myocarditis/myopericarditis was based on symptoms (chest discomfort 11/12, dyspnea 3/4), elevated troponin (range 165-21200 ng/L, peaking 1-2 days after cardiac symptom onset), supportive electrocardiogram (ECG) findings (diffuse or territorial ST elevation, T-wave inversions, and/or non-specific ECG changes 9/12), and/or cardiac imaging findings (pericardial effusion 1/12, left ventricle [LV] abnormalities on echocardiogram 4/12, abnormal cardiac MRI in 7/7 done acutely). In the pericarditis case, ECG showed widespread ST elevation and echocardiogram showed hyperdynamic LV. Treatments included ASA or non-steroidal anti-inflammatory drugs (n=7), tecovirimat (n=5), colchicine (n=4), ACE-inhibitors (n=3) and bisoprolol (n=3). All were hospitalized, with lengths of stay of 4-10 days, and at least 3 patients required intensive care. Cardiac symptom recovery occurred within 1-3 days of admission; in at least 1 patient symptoms continued beyond 1 month.

**Conclusion:**

Mpox is rarely associated with myocarditis and/or pericarditis, with cardiac symptoms beginning on day 2-8 after illness onset. Long-term outcomes require further study.

**Disclosures:**

**Cécile Tremblay, MD**, Association canadienne de protection médicale: Expert Testimony|Astra-Zeneca: Advisor/Consultant|Astra-Zeneca: Honoraria|Canadian Institutes of Health Research: Grant/Research Support|Gilead: Advisor/Consultant|Gilead: Grant/Research Support|Gilead: Honoraria|GlaxoSmithKline: Advisor/Consultant|GlaxoSmithKline: Honoraria|Medicago: Advisor/Consultant|Merck: Advisor/Consultant|Merck: Grant/Research Support|Merck: Honoraria|National Institute of Health: Grant/Research Support|Sanofi: Advisor/Consultant **Darrell H. S. Tan, MD PhD**, Abbvie: Grant/Research Support|Gilead: Grant/Research Support|Glaxo Smith Kline: Grant/Research Support

